# Improved Osseointegration of Selective Laser Melting Titanium Implants with Unique Dual Micro/Nano-Scale Surface Topography

**DOI:** 10.3390/ma15217811

**Published:** 2022-11-05

**Authors:** Xuetong Sun, Huaishu Lin, Chunyu Zhang, Ruiran Huang, Ying Liu, Gong Zhang, Si Di

**Affiliations:** 1 Center for Precision Engineering, Guangzhou Institutes of Advanced Technology, Guangzhou 511458, China; 2 Guangdong Technical College of Water Resources and Electric Engineering, Guangzhou 510925, China; 3 Guangzhou Janus Biotechnology Co., Ltd., Guangzhou 511400, China

**Keywords:** titanium, mesoporous bioactive glass, micro/nano- topography, bone implants, selective laser melting

## Abstract

Selective laser melting manufacture of patient specific Ti implants is serving as a promising approach for bone tissue engineering. The success of implantation is governed by effective osseointegration, which depends on the surface properties of implants. To improve the bioactivity and osteogenesis, the universal surface treatment for SLM-Ti implants is to remove the primitive roughness and then reengineer new roughness by various methods. In this study, the micro-sized partially melted Ti particles on the SLM-Ti surface were preserved for assembling mesoporous bioactive glass nanospheres to obtain a unique micro/nano- topography through combination of SLM manufacture and sol–gel processes. The results of simulated body fluid immersion test showed that bioactive ions (Ca, Si) can be continuously and stably released from the MBG nanospheres. The osseointegration properties of SLM-Ti samples, examined using pre-osteoblast cells, showed enhanced adhesion and osteogenic differentiation compared with commercial pure titanium commonly used as orthopedic implants. Overall, the developed approach of construction of the dual micro/nano topography generated on the SLM-Ti native surface could be critical to enhance musculoskeletal implant performance.

## 1. Introduction

Titanium and its alloys are considered one of the most widely used metals in orthopedic implants due to the good corrosion resistance and biomechanical compatibility [1,2]. However, the biological inertness of Ti implants may compromise its early stability and long-term success, especially in complicated clinical cases typically caused by traumatic injuries [3,4]. Personalized bone implants with adjustable biomechanical properties and optimized topology design are possible due to 3D printing technology [5,6,7]. In recent years, 3D printed metal techniques have played an increasingly important role in the application of orthopedic implantation. Among them, selective laser melting (SLM) has drawn increasing attention from both medical and industrial communities [7,8].

The SLM technique has been extensively used to fabricate titanium implants with complex geometries and precise construction. Despite the promising advantages of the SLM technique, the surface microstructure of SLM-built titanium implants is quite different from that of traditional powder metallurgy titanium implants [9,10]. The SLM technique produces parts by employing a focused and computer-controlled laser to melt metal particles together at a rapid rate. The primitive surface of SLM parts is covered with numerous partially melting powder particles as a result of the rapid cooling [11]. The mechanical properties of SLM-Ti parts with complex-shapes or porous structures are in agreement with the application standard for bone replacement materials [12,13]. Implant surface microstructure is one of key factors in the process of bone formation at the implant–tissue interface, especially for biologically inert titanium alloy [14,15]. There is increasing evidence that controlling the surface roughness and wettability of implants can have an effect on the attachment, proliferation and differentiation of osteoblast-like cells [16,17].

Previous studies have shown whether removal of micron-sized Ti particles from SLM-Ti surface is beneficial for osseointegration remains a controversial subject. Takemoto et al. [18] suggested that although the microrough SLM-Ti surface could be conducive to the initial mechanical stability, it was ineffective for osteogenic differentiation and should be performed with a bioactive modification to promote the bone regeneration. Hao et at. [19] found that the process-inherent Ti particles on SLM-Ti porous scaffolds not only enhanced bacterial adhesion, but also inhibited the osteogenic ability of hBMSCs. However, Deng et al. [20] demonstrated that the microrough surface of SLM-Ti implants were conducive to the mechanical interlock in the initial osseointegration. Chen et al. [21] found that the primitive surface of SLM-built scaffolds favors cell adhesion and migration due to micro- scale particles and ravines. Thus, the SLM-Ti implants with the primitive surface topography may be beneficial for early mechanical stability, but also carry the risk of inducing bacterial adhesion.

In addition, one of the most unique features of 3D printed personalized implants is the free design of complex shapes and interconnected porous structures [22,23]. The inner surface of porous hip implants, knee implants and artificial vertebrae is a challenge for several surface treatment methods, such as mechanical machining, because they can either be unreachable to the ‘deep hole’, jam up the interconnected pores or even introduce cytotoxicity. Therefore, the effect of micro-sized Ti particles of SLM-Ti native surface on osseointegration is an unavoidably important subject. However, the current research on 3D printing of bone implants is mainly focused on the polished surface topography through post-manufacture.

Based on previous studies, the dual micro- and nano-scale topography of the titanium implants promotes bone formation through the synergistic functions [24,25]. The micro-topography affects the early cell adhesion and proliferation, while the nano-topography regulates protein conformation and further modulates osteoblast differentiation [26,27,28,29]. It is noteworthy that titanium implants with micro- and nano- roughness characteristics were commonly prepared through mixed surface modification methods (sand-blasting, acid etching). The regular surface treatment for SLM-Ti implants is first to remove the undesired micro-Ti particles as a pre-treatment, then to construct the new micro- structure and further to construct nano- structure as a reengineering treatment [30,31,32]. Limited studies have focused on the inner surface of porous SLM-Ti implants, which can only retain the partially melted powder particles.

Thus, the aim of this work was to develop a novel surface modification method to fabricate SLM-Ti implants with unique dual micro- and nano-topography, which was designed by a combination of micro-Ti particles and mesoporous bioactive glasses (MBG) nano-spheres. The MBG nanospheres with particle size of 300–400 nm were synthesized by the sol–gel method [33,34,35]. Bioactive glasses can continuously and rapidly release Ca, P and Si ions and induce the formation of hydroxyapatite layer in stimulate body fluid [36,37,38,39]. The dual scale MBG-SLM-Ti surface and single nanoscale MBG-CP-Ti surface were compared in wettability, ions release profile and in vitro cell experiments in terms of adhesion, proliferation and differentiation of osteoblast-like cells. The motivation of this study is to provide an option for 3D printed implants with complex shapes and porous structures to improve the osseointegration performance by retaining micron-size Ti particles for construction of micro-/nano- topography, which will help to reduce post printing modifications.

## 2. Material and Methods

### 2.1. Specimens Preparation

The CP-Ti foils (purity > 99.7%, Ф13 mm × 1 mm) were purchased from Baoji Titanium Industry Co., Ltd. (Shanxi, China). SLM-Ti discs (Ф13 mm × 1 mm) were fabricated with a Concept laser Mlab 200R machine (Concept laser, Lichtenfels, Germany), equipped with a 200 W Yb YAG fiber laser. Commercial spherical pure titanium powder (>99.5% purity) with a particle size distribution between 20 and 40 μm was used. A laser power of 200 W, a scanning speed of 400 mm/s, a power layer thickness of 25 μm, a hatch distance of 60 μm and a spot size of 75 μm were selected to control the SLM process. The building chamber was first vacuumized and then filled with the argon atmosphere. Once the build process was finished, excess powder was removed from the sample surfaces. In addition, the sample surface was maintained in the rough as-built condition without additional post-treatment.

### 2.2. Formation of MBG Coatings

An amount of 2 g cetyltrimethylammonium bromide (CTAB) was dissolved in 104 mL ethyl alcohol and deionized water with stirring, after that 4 mL ammonia solution was added into the solution. After stirring for 15 min at 40 °C, 4 mL tetraethyl orthosilicate (TEOS), 0.31 mL triethylphosphate (TEP) and 0.82 g calcium nitrate tetrahydrate (CN) were sequentially added in 30 min intervals under stirring. The MBG nanoparticles was collected through centrifugation after 3 h reaction at 40 °C. After rinsing with ethyl alcohol three times, the MBG nanoparticles were used to prepare MBG slurry with ethyl alcohol in different concentrations. SLM-Ti and CP-Ti substrates were dipped into MBG slurry for 3 and 7 cycles and dried at room temperature, after which they were calcinated at 650 °C for 3 h at a heating rate of 2 °C/min.

### 2.3. Surface Characterization

The surface morphology of samples was characterized by a field emission scanning electron microscope (Hitachi SU8220, Japan). Leica DVM6 digital video microscope was used for optical 3D profile analysis. ImageJ software was used to analyze the particles size and the size distribution is plotted. Surface wettability of samples was evaluated by a static contact angle goniometer (SDC-100 system Sindin Products, Dongguan China). The sessile-drop method was used to determine the water contact angles with a dose of each droplet of 4 μL at the room temperature of 25 ± 3 °C and the relative humidity of 65 ± 5%. Before the measurements, the specimens were rinsed by distilled water and dried in air.

### 2.4. In Vitro Bioactivity

The in vitro bioactivity of samples was determined by the immersing in SBF solution at 37 °C for 7 days. The vessels were placed in an orbital shaker for 7 days at a speed of 180 r/min and temperature of 37 °C. At each selected time point (1, 3 and 7 days), the samples were rinsed with distilled water and dried at room temperature. Apatite formation and elemental composition were characterized using SEM equipped with X-ray energy dispersion spectroscopy (EDS). Meanwhile, the concentration of Si and Ca ions released from MBG-CP-Ti and MBG-SLM-Ti were measured by an inductively-coupled plasma atomic emission spectrometry (ICP-AES; Varian Co., Palo Alto, CA, USA).

### 2.5. In Vitro Biological Evaluations

#### 2.5.1. Cell Culture

The mouse osteoblastic precursor cells (MC3T3-E1) were supplied by the Stomatological Hospital of Sun Yat-sen University. All samples were disinfected by autoclaving, immersed in pure ethanol for 4 min and dried under UV light. The sterilized samples were inoculated with 30,000 osteoblastic cells and cultured in α-minimum essential medium (α-MEM) containing 10% fetal bovine serum (FBS) and 1% penicillin–streptomycin solution in a humidified atmosphere of 5% CO_2_ at 37 °C. The culture medium was renewed every 2 days during the entire cell-culturing period. The MC3T3s at passage 3–4 were employed to conduct the following cell experiments.

#### 2.5.2. Cell Imaging

The cell morphologies were observed by a field emission scanning electron microscope (Hitachi SU8220, Tokyo, Japan) with a secondary electronic resolution (1.1 nm/1 kV, 0.8 nm/15 kV) and a laser confocal microscopy (Leica TCS SP8) with a confocal resolution: XY ≤ 140 nm, Z ≤ 400 nm. MC3T3-E1 cells were incubated on the specimen surfaces in 24-well plates at a density of 3 × 10^4^ cells mL^−1^ for 72 h. For SEM imaging, the cells were fixed in 2.5% glutaraldehyde for 6 h, sequentially dehydrated in ethanol solutions (50%, 75%, 80%, 90% and 100%) and air-drying. For confocal fluorescence microscopic imaging, the F-actin and nuclei were stained with Actin-Tracker Red-549 for 45 min and Hoechst 33258 for 15 min in darkness, respectively.

#### 2.5.3. Cell Proliferation

CCK-8 assay was carried out to investigate the MC3T3-E1 proliferation. After being cultured for 1, 3 and 7 days, the adhered cells were digested from the samples and incubated in fresh medium containing 10% CCK-8 solution for 4 h. Then, the optical density (OD) values were determined at 450 nm using an enzyme-linked immunosorbent assay plate reader (SMR, Biotek, Winooski, VT, USA).

#### 2.5.4. Cell Differentiation

The early stage of cell differentiation on samples was determined by alkaline phosphatase activity (ALP) assay and bicinchoninic acid (BCA) protein assay kit. After being cultured for 7 and 14 days, the cells on samples were washed with sterile PBS and treated with 0.1% Triton X-100 lysis buffer for 30 to 40 min. Then, the lysate was centrifuged and the supernatant as used to carried out ALP activity assay according to a commercially available ALP assay kit’s instructions. The ALP activity was normalized with respect to the total protein content obtained from the same cell lysate measured by the BCA Kit.

### 2.6. Statistical Analysis

All experimental data were analyzed as mean ± standard deviation from at least five separate experiments for each variable. The difference was expressed by GraphPad Prism (GraphPad, CA, USA). The significant level of statistical analysis was set as * *p* < 0.05, and a value of ** *p* < 0.01 was considered to be significantly different.

## 3. Results and Discussion

### 3.1. Surface Topography of SLM-Ti

**Figure 1:**
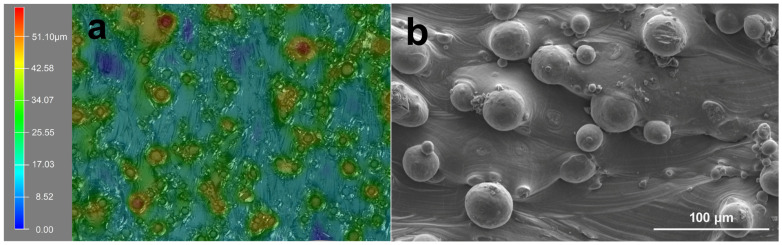
Representation of (**a**) optical surface profiles and (**b**) SEM morphology of SLM-Ti substrate.

The optical 3D surface profiles and SEM morphologies of primitive SLM-Ti surface are shown in Figure 1. Both images show that the SLM-Ti native surface is covered with a large number of micro-sized spherical Ti particles. The diameters of the Ti particles were mainly distributed from 20 to 40 μm, as depicted in Figure 1a,b. These particles are metallurgically connected to the underlying SLM-Ti surface and were dispersed from each other, although a few interconnected micro-particles were also observed (Figure 1b). The average inter-particle distance ranges from 20 μm to 100 μm.

### 3.2. Morphology of MBG Coated SLM-Ti and CP-Ti Surfaces

**Figure 2:**
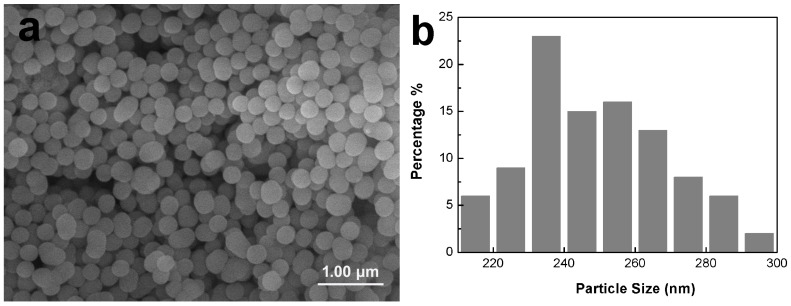
Morphology (**a**) and particle size distribution (**b**) of MBG nanospheres fabricated by sol–gel.

**Figure 3:**
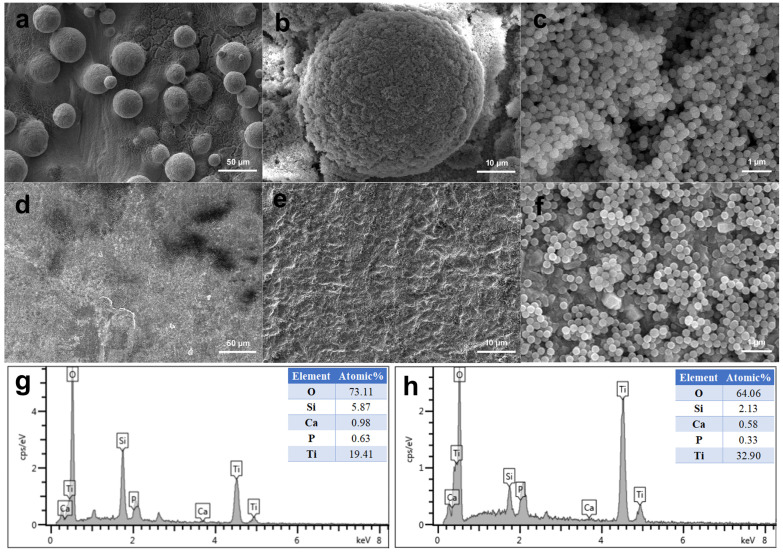
Morphologies of MBG-coated SLM-Ti (**a**–**c**) and CP-Ti (**d**–**f**) by dip coating, and EDS analysis of MBG-SLM-Ti (**g**) and MBG-CP-Ti (**h**).

As shown in Figure 2, the MBG nanospheres were fabricated through the sol–gel method with a narrow particles size distribution between 230 nm and 300 nm. The surface morphologies of MBG-coated SLM-Ti and CP-Ti by dip coating are presented in Figure 3. It can be seen that MBG nanoparticles are homogeneously distributed on SLM-Ti and CP-Ti surfaces. For MBG-coated SLM-Ti, its surface is a dual scale topography composed of the micron-sized spherical Ti particles and the nano-sized spherical MBG particles. The number of MBG particles coated on SLM-Ti is higher than that coated on CP-Ti (Figure 3a–f), because the dip coating thickness of the gel accommodated in the ‘valleys’ on SLM-Ti surface is thicker than that on the relatively smooth CP-Ti surface without micro-sized Ti particles.

EDS elemental analysis was performed on MBG-SLM-Ti and MBG-CP-Ti surfaces presented in Figure 3. EDS analysis revealed that the detected coated elements on both surfaces were mainly silicon, calcium, phosphorus and oxygen as the substrate element was titanium. The ratio of Si, Ca and P on both surfaces was about 80:15:5, which was in line with the expected result of the sol composition.

### 3.3. Surface Wettability of MBG Coated SLM-Ti and CP-Ti

**Figure 4:**
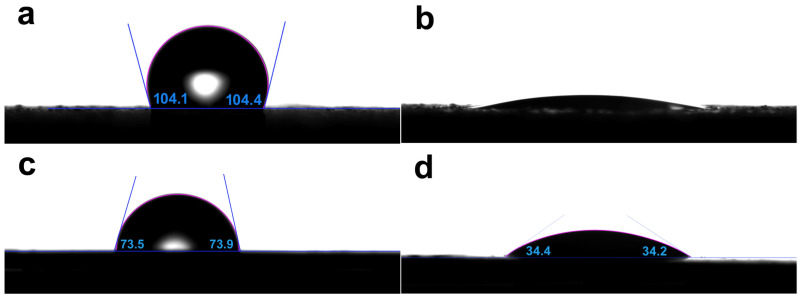
Water contact angles of (**a**) SLM-Ti, (**b**) MBG-SLM-Ti, (**c**) CP-Ti and (**d**) MBG-CP-Ti, showing the difference in wettability.

To characterize the effect of the surface wettability on the bioactivity of MBG coated SLM-Ti and CP-Ti surfaces, their water contact angles (WCAs) were measured. As shown in Figure 4, the WCAs of SLM-Ti and CP-Ti are 104.1 ± 2.5° and 73.7 ± 2.5°, respectively, while the WCAs of MBG- SLM-Ti and MBG- CP-Ti are reduced to < 5° and 34.3 ± 2.5°, respectively. For SLM-Ti and CP-Ti surfaces with the same chemical composition, it is hypothesized that the process-inherent roughness changes the surface from hydrophilic (CP-Ti) to hydrophobic (SLM-Ti). In previous studies, the surface hydrophobicity is one of the main factors in removing the primitive micro-sized Ti particles of SLM-Ti implants.

The apparent contact angle of a liquid droplet on a low roughness surface can commonly be explained by Wenzel’s model [40]. According to the Wenzel equation, increasing surface roughness will make a hydrophilic surface more hydrophilic and a hydrophobic surface more hydrophobic [41,42]. Therefore, the MBG-CP-Ti showed stronger hydrophilicity than the CP-Ti. However, on a higher roughness surface, air or droplets can be entrapped, and, thus, a composite contact interfaces occurs according to Cassie–Baxter’s model [43,44]. For the MBG-SLM-Ti surface, a large number of MBG nanoparticles accommodated in the ‘valleys’ between micro-spherical Ti particles. When a droplet is dropped onto the MBG-SLM-Ti surface, a portion of this can be sucked into the MBG nanoparticles in the ‘valleys’ and the rest is dropped onto the mixture surface of the droplet and nanoparticles. Thus, SLM-Ti surface changes from hydrophobic to superhydrophilic after coating with MBG nanospheres. Surface wettability can commonly be used to indirectly estimate the cell proliferation capacity [45,46] since cells prefer hydrophilic surfaces to hydrophobic ones. Thus, MBG nanosphere coatings may increase osteoblasts proliferation on SLM-Ti and CP-Ti surfaces, especially for SLM-Ti surface.

### 3.4. In Vitro Bioactivity

**Figure 5:**
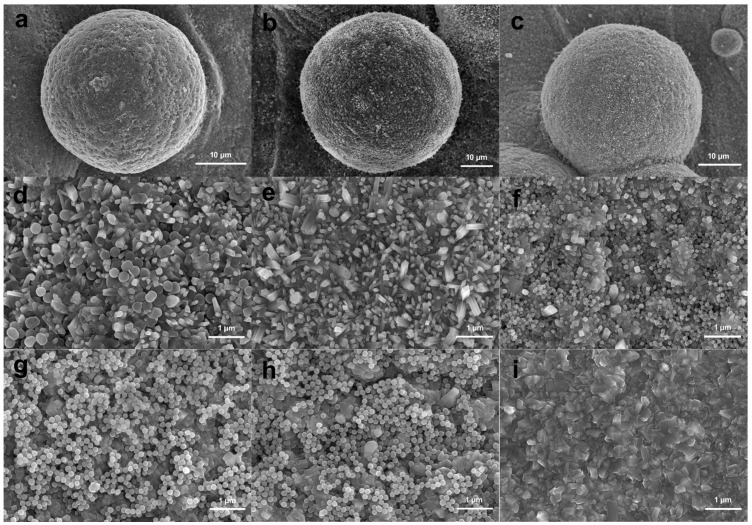
SEM surface morphology of MBG-SLM-Ti (**a**–**f**) and MBG-CP-Ti (**g**–**i**) after immersion in SBF for 1 (**a**,**d**,**g**), 3 (**b**,**e**,**h**) and 7 (**c**,**e**,**i**) days.

**Figure 6:**
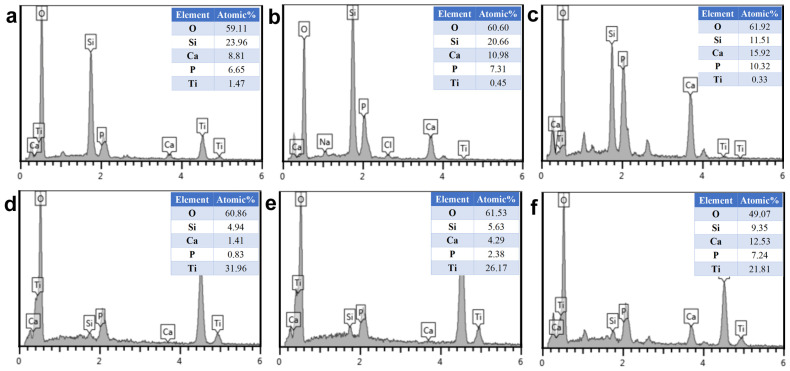
EDS analysis of MBG-SLM-Ti (**a**–**c**) and MBG-CP-Ti (**d**–**f**) after immersion in SBF for 1 (**a**,**d**), 3 (**b**,**e**) and 7 (**c**,**f**) days.

In vitro bioactivity of the MBG-SLM-Ti and MBG-CP-Ti was evaluated in terms of apatite mineralization after the SBF immersion. Figure 5 shows SEM images of MBG-SLM-Ti and MBG-CP-Ti surfaces after different SBF immersion intervals. After 24 h of SBF immersion, the rod-like particles (100 nm in length) were observed on MBG-SLM-Ti surface (Figure 5d), while MBG-CP-Ti surface was almost unchanged (Figure 5g). The morphology of the rod-like particles was similar to that of hydroxylcarbonate apatite in human bones [44]. It took 72 h for a layer of rod-like particles to cover all the MBG-SLM-Ti surface (Figure 5e), compared with 7 days for MBG-CP-Ti surface (Figure 5i). EDS analysis (Figure 6) revealed that the Ca, P and Si contents of MBG-SLM-Ti increased with increased immersion time, while those of MBG-CP-Ti did not change significantly.

**Figure 7:**
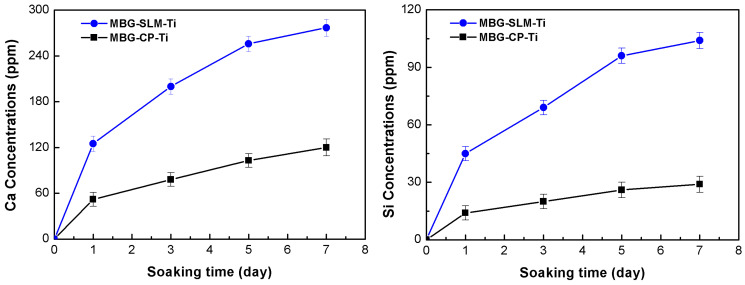
Release profiles of Ca and Si ions from the MBG-SLM-Ti and MBG-CP-Ti after soaking in the cell culture solution for 1, 3, 5 and 7 days.

Figure 7 presents the variations of Si and Ca ions concentrations released from the MBG-SLM-Ti and MBG-CP-Ti after immersion in the cell culture medium, as a function of the time. The release patterns of Si and Ca ions from the MBG-SLM-Ti and MBG-CP-Ti is quite similar, while the concentration of Ca ions was obviously higher than that of Si ions and the difference increased gradually with immersion time. Notably, MBG-SLM-Ti released more Ca and Si ions than MBG-CP-Ti, reaching 104 ppm Si and 277 ppm Ca on day 7. This result confirmed that the dual scale micro- and nano- topography of MBG-SLM-Ti could continuously and steadily release Ca and Si ions due to the extremely high specific surface area of MBG-SLM-Ti. The sustained release of bioative Ca and Si ions can provide continuous nutrients for cells proliferation and long-term osteogenesis [47,48].

### 3.5. Cell Morphology

**Figure 8:**
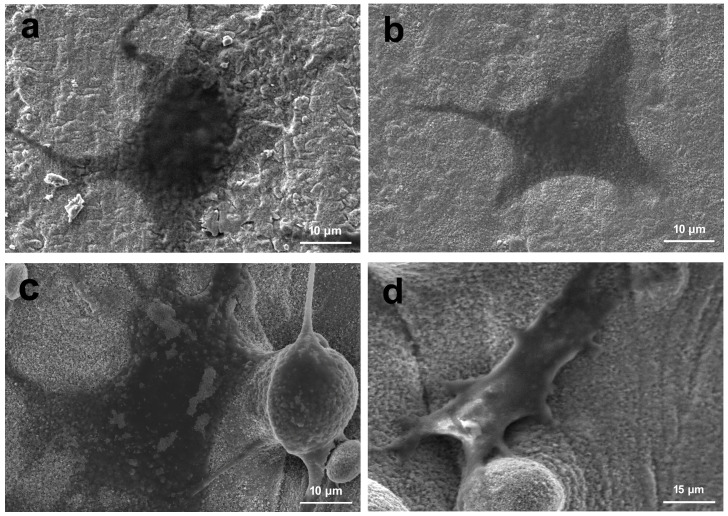
SEM images of MC3T3-E1s cell cultured on the (**a**,**b**) MBG-SLM-Ti and (**c**,**d**) MBG-CP-Ti after 3 day.

SEM images of MC3T3-E1 cell morphology after 3 days of culture on MBG-SLM-Ti and MBG-CP-Ti surfaces are presented in Figure 8. On the MBG-CP-Ti, the cells can fully spread and display a stretched polygonal shape Figure 8a,b. On the MBG-SLM-Ti, the cells were focally attached to the micro-scale ‘valleys and peaks’ surface, in combination with MBG nanoparticles in a three-dimensional orientation Figure 8c,d. Osteoblastic extensions were observed between the individual micro-spherical particles with evidence of dendritic extensions (Figure 8c).

**Figure 9:**
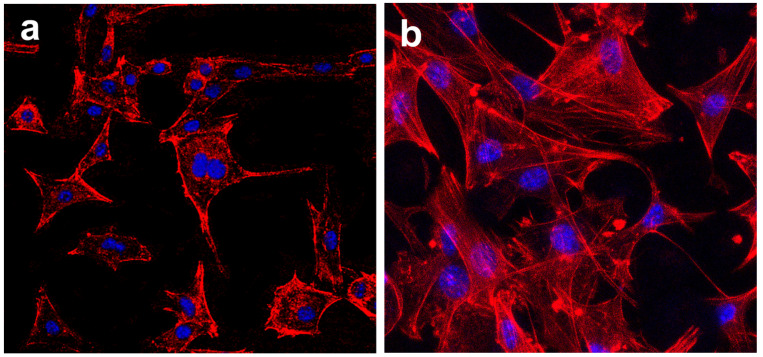
CLSM (red (cytoskeleton) and blue (nuclei)) of MC3T3s cell on (**a**) MBG-CP-Ti and (**b**) MBG-SLM-Ti after 3 days.

Confocal imaging after 3 days incubation also presented cells attaching and spreading on samples surfaces. As shown in Figure 9, the cell morphology differed greatly on MBG-SLM-Ti and MBG-CP-Ti surfaces. On the MBG-CP-Ti, cells were attached in a two-dimensional orientation with pseudopodia. On the MBG-SLM-Ti surface, cells showed many interlaced filopodia-like extensions and distinct stress fibers, suggesting a strong cellular anchoring on the dual-scale structures.

### 3.6. Cell Viability Evaluation

**Figure 10:**
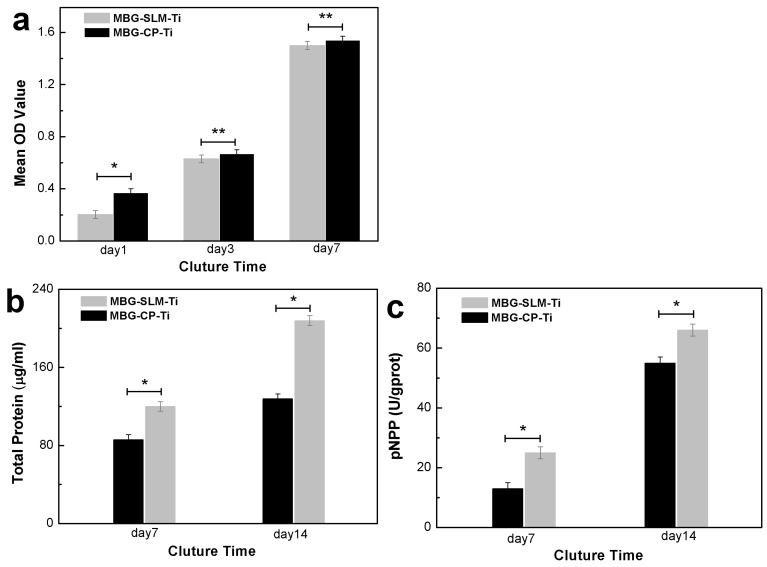
Cell viability evaluated through (**a**) the cell proliferation rates after 1, 3 and 7 days, (**b**) total protein contents and (**c**) ALP activity after 7 and 14 days. (* *p* < 0.05, ** *p* < 0.05).

The results of the CCK-8 assay of MC3T3s cultured on the MBG-SLM-Ti and MBG-CP-Ti surface for 1, 3 and 7 days are presented in Figure 10a. After culturing for 1 day, the proliferation rates of MC3T3s on MBG-SLM-Ti were slightly lower than those of MBG-CP-Ti. It can be speculated that it may be easier for cells to adhere in the flat CP-Ti surface. By day 3 and 7, however, the cell proliferation levels of the MBG-SLM-Ti and MBG-CP-Ti became nearly the same. Cell differentiation was examined by ALP activities and the total protein contents after 7 and 14 days of cells culture, and the results are shown in Figure 10b,c. An obvious higher ALP activity and total protein contents were observed for MBG-SLM-Ti after culturing for 7 and 14 days compared to MBG-CP-Ti. Previous studies have shown that the native SLM-Ti surface performed poorly in terms of cell proliferation and osteoblast differentiation [19]. In this work, MC3T3s cultured on the MBG-SLM-Ti and MBG-CP-Ti surfaces showed similar cell proliferation rates. Cell morphology and cytoskeletal tension are important in determining osteogenesis of osteoblast. For cells on the MBG-SLM-Ti surface, stress fibers, as the cytoskeletal responses to mechanical stimulation [49], could be observed, which suggests this dual micro- and nano- topography has the force-generating nature. In addition, the MBG-SLM-Ti surface exhibited higher total protein contents and ALP activity than MBG-CP-Ti on both 7th and 14th day. Nanotopography is commonly considered to promote osteogenic differentiation through modulating protein conformation and signal transduction. Compared with the smooth MBG-CP-Ti, the MBG-SLM-Ti with numerous micro-scale ‘peaks and valleys’ has more MBG nanospheres. This indicates that the bioactive MBG nanospheres, which modified the surface topography and physicochemical properties of SLM-Ti, can regulate its osteoblast morphology and differentiation state.

## 4. Conclusions

In this work, a novel approach has been developed to modify SLM-Ti surface with enhanced osteogenesis and osseointegration properties by forming the dual micro- and nano-topography composed of micro-scale Ti particles and MBG nanospheres. The micro-sized Ti particles on primitive SLM-Ti surfaces was preserved. MBG nanospheres were fabricated by a sol–gel process on SLM-Ti surface including micro-particles.

With the presence of MBG nanospheres, the initial hydrophobic surface of SLM-Ti was altered into superhydrophilic, while the hydrophilic surface of CP-Ti surface was altered into more hydrophilic. MBG-SLM-Ti could readily and continuously release more bioactive Ca and Si ions into the surrounding cell culture solution due to its higher surface areas, which could provide sufficient nutrients for cells. The in vitro cell culture experiments showed that MBG-SLM-Ti performed better in terms of cell adhesion, proliferation and differentiation compared to MBG-CP-Ti. As such, this dual micro-/nano- topography has great potential bespoke implants applications.

This innovative approach offers a cost-effective and scalable tool to optimize additive manufacturing processes and simplify the surface modification processes. Ultimately, this tool can be used to improve the osseointegration performance of SLM-Ti implants with complex shapes and porous structures by retaining micron-size Ti particles for construction of micro-/nano- topography. However, this work does not involve the infection rates of SLM-Ti implants, which will be the focus of the future work.

## Data Availability

The data that support the findings of this study are available from the corresponding author upon reasonable request.
